# TME6/365: Teleconsultation and Telescreening for Eye Diseases

**DOI:** 10.2196/jmir.1.suppl1.e113

**Published:** 1999-09-19

**Authors:** G Zahlmann, G Mann

**Affiliations:** ^1^GSF - MEDIS, NeuherbergGermany

**Keywords:** Teleconsultation, Telemedicine, Telescreening

## Abstract

**Introduction:**

The estimated prevalence of diabetes mellitus is 6.6 % of the population in Western industrial countries. Approximately 85% of the population eventually develop a diabetic retinopathy (DR). About half of the patients with the severe form of DR - proliferative DR will become blind within 5 years. Blindness can be prevented by an early treatment avoiding severe status. Screening diabetes patients or even the general population at risk can make the difference between blindness and cure. Telescreening is one approach to establish such a service. Cataract is an eye disease, mainly of the ageing population, changing the properties of the eye lens by becoming opaque. Replacing this lens by an artificial one is the current approach to treatment. This surgery gives back the patients their visual acuity before the lens became opaque. Pre- and postoperative care is given by private ophthalmologists. The surgery itself is performed in hospitals or by other private surgeon-ophthalmologists. Therefore the patient has to visit two different ophthalmologists. Getting all partners within this process together by means of telecommunication solutions can reduce the effort and strengthen the communication and co-operation.

**Methods:**

A telescreening system for DR has been developed. Two 45° fundus images per eye are taken by a Topcon fundus camera with a dilated pupil. The digital images (Topcon image net) are sent together with additional data about the patient to graders. Special software based on Internet standards has been developed combining images and other data in one report. Investigating perioperative cataract - OP - management a combination of synchronous and asynchronous teleconsultation has been realized using PGP-encrypted Internet e-mail and commercially available videoconferencing equipment.

**Results:**

Regarding telescreening two studies have been performed. The first multi-center study dealt with diagnostic accuracy of the telescreening solution versus a gold standard. The quality of the telescreening has been proven as high enough to apply this technology for screening of diabetes patients. A second study was about the diagnostic accuracy of the approach to detect macular edema. The perioperative cataract - OP - management has been accepted by the patients. Their effort has been reduced by one physician's visit. The co-operation between the ophthalmologists and the surgeon was improved.

**Discussion:**

Both teleconsultation applications show their feasibility and applicability in the domain of ophthalmology. Both are prototype solutions. The next steps have to be a broader application and well-defined outcome studies to make it real-life services.

